# PGM5-AS1 impairs miR-587-mediated GDF10 inhibition and abrogates progression of prostate cancer

**DOI:** 10.1186/s12967-020-02572-w

**Published:** 2021-01-06

**Authors:** Lei Du, Yongli Gao

**Affiliations:** Department of Oncology, Linyi People’s Hospital, No. 27, East Section of Jiefang RoadShandong, Linyi, 276000 People’s Republic of China

**Keywords:** Prostate cancer, PGM5-AS1, MicroRNA-587, GDF10, Proliferation, Apoptosis

## Abstract

**Background:**

Prostate cancer (PCa) is a leading cause of cancer-related death in males. Aberrant expression of long non-coding RNAs (lncRNAs) has been implicated in various human malignancies, including PCa. This study aims to clarify the inhibitory role of human PGM5 antisense RNA 1 (PGM5-AS1) in the proliferation and apoptosis of PCa cells.

**Methods:**

The regulatory network of PGM5-AS1/microRNA-587 (miR-587)/growth and differentiation factor 10 (GDF10) axis was examined by dual-luciferase reporter gene assay, RNA-binding protein immunoprecipitation, and RNA pull down assay. We manipulated the expression of PGM5-AS1, miR-587 and GDF10 by transducing expression vectors, mimic, inhibitor, or short hairpin RNA into PCa cells, thus establishing their functions in cell proliferation and apoptosis. Additionally, we measured the tumorigenicity of PCa cells xenografted in nude mice.

**Results:**

PGM5-AS1 is expressed at low levels in PCa cell lines. Forced overexpression of PGM5-AS1 restricted proliferation and facilitated apoptosis of PCa cells, manifesting in suppressed xenograft tumor growth in nude mice. Notably, PGM5-AS1 competitively bound to miR-587, which directly targets GDF10. We further validated that the anti-cancer role of PGM5-AS1 in PCa cells was achieved by binding to miR-587 to promote the expression of GDF10.

**Conclusion:**

PGM5-AS1 upregulates GDF10 gene expression by competitively binding to miR-587, thus inhibiting proliferation and accelerating apoptosis of PCa cells.

## Background

Prostate cancer (PCa) is one of the most common causes of cancer death in males across the world [1]. Based on an Annual Report to the Nation on the Status of Cancer by Negoita et al*.*, new PCa cases identified by prostate-specific antigen testing underwent a decrease from 2008 onwards, whereas the incidence of late stage diagnosis rose from 2010 onwards [2]. The importance of PSA screening is a matter of debate, given that current PCa screening and treatment protocols can lead to clinical harm, such as infection and urogenital side effects due to overdiagnosis and overtreatment [3, 4]. However, the 5-year survival rate approaches 100% for patients with localized PCa, but is only 28% for those with distant metastasis [5]. The obstacles due to current standards of PCa detection and therapy motivate researchers to probe the molecular mechanism underlying the pathophysiology of prostate carcinoma so as to identify better non-invasive biomarkers for early diagnosis and treatment.

There is a paucity of evidence demonstrating the involvement of long non-coding RNAs (lncRNAs) in pathogenesis and metastasis of PCa, despite indications that lncRNA dysregulation has a link with PCa progression [6]. LncRNAs represent a distinctly heterogeneous family of RNA transcripts with a length over 200 nucleotides exhibiting little or no coding potential; the expression patterns and mechanisms of lncRNAs in PCa are a subject of active investigation to identify possible diagnostic and therapeutic strategies [7]. Interestingly, identification of regulatory network of interrelated lncRNAs, microRNAs (miRNAs) and messenger RNAs (mRNAs) provides new insight into the molecular mechanisms underlying tumorigenesis of PCa and its development [8]. In this study, our preliminary bioinformatics analysis first identified the involvement of the human PGM5 antisense RNA 1 (PGM5-AS1)/miR-587/growth and differentiation factor 10 (GDF10) axis in the malignant phenotypes of PCa.

Prior evidence has proposed a tumor suppressive role of PGM5-AS1 in colorectal cancer, and its ectopic expression induces cell apoptosis and cell cycle arrest in colorectal cancer [9]. Furthermore, the lncRNA-miRNA-mRNA co-expression network has been suggested to explain the function of PGM5-AS1 through its sequestration of miR-466 to elevate gene expression of the phosphate and tension homology deleted on chromosome ten (PTEN), thereby inhibiting esophageal squamous cell carcinoma progression [10]. In terms of the predicted binding between site miR-587 and PGM5-AS1, its overexpression was documented to abrogate 5-fluorouracil-induced apoptosis of colorectal cancer cells and impede the inhibition of tumor growth, which was realized by inversely regulating the target gene PPP2R1B [11]. Moreover, the predicted target of miR-587 in this study, GDF10, also known as BMP3B, belongs to the transforming growth factor-β (TGF-β) family [12], which has been indicated as a tumor suppressor in lung cancer and also a factor in the progression of PCa [13]. Accordingly, we tested in the study functional relevance of PGM5-AS1/miR-587/GDF10 axis in PCa cells and xenografts tumor.

## Methods and materials

### Ethics statement

The study protocols were approved by the Institutional Animal Care and Use Committee of Linyi People's Hospital. The animal experiments were performed in strict accordance with the recommendations in the Guide for the Care and Use of Laboratory Animals of the National Institutes of Health.

### Microarray-based gene expression profiling

The PCa-related microarray datasets (GSE3325 and GSE30994) and probe annotation files were downloaded from the Gene Expression Omnibus database. We conducted differential expression analysis to retrieve differentially expressed genes (DEGs) with the assistance of Limma package of R software. |logFoldChange|> 2 and *p* value < 0.05 were set as the thresholds and a heat map for DEGs was plotted by pheatmap package.

### Cell treatment

The human PCa cell lines (PC-3, LNCap, 22RV1 and DU145) and normal prostatic epithelial cell line RWPE-1 were obtained from American Type Culture Collection (Manassas, VA, USA). BPH1 cells were purchased from Cell Bank of the Chinese Academy of Sciences (Shanghai, China). Following rapid recovery, these cells were incubated in Roswell Park Memorial Institute (RPMI) 1640 medium (11875119, Gibco Life Technologies, Grand Island, NY, USA) containing 10% fetal bovine serum (10099141, Gibco Life Technologies). Then, cells were further cultured in medium supplemented with 100 U/mL penicillin and 100 U/mL streptomycin at 37 °C in a 5% CO_2_ incubator. When cells achieved 80% confluence, they were trypsinized.

The cells were assigned to ten treatment groups as follows: (1) blank (cells without any treatment), (2) negative control for overexpression plasmid (oe-NC) (cells transduced with NC of overexpression plasmid), (3) oe-PGM5-AS1 (cells transduced with plasmid overexpressing PGM5-AS1), (4) inhibitor-NC + sh-NC (cells transduced with NC of miR-587 inhibitor and NC of short hairpin RNA [shRNA] against GDF10), (5) miR-587 inhibitor + sh-NC (cells transduced with miR-587 inhibitor and NC of shRNA against GDF10), (6) miR-587 inhibitor + sh-GDF10 (cells transduced with miR-587 inhibitor and shRNA against GDF10), (7) oe-PGM5-AS1 + mimic-NC (cells transduced with plasmid overexpressing PGM5-AS1 and NC of miR-587 mimic), (8) oe-PGM5-AS1 + miR-587 mimic (cells transduced with plasmid overexpressing PGM5-AS1 and miR-587 mimic), (9) oe-PGM5-AS1 + sh-NC (cells transduced with plasmid overexpressing PGM5-AS1 and sh-NC), and (10) oe-PGM5-AS1 + sh-GDF10 (cells transduced with plasmid overexpressing PGM5-AS1 plasmid overexpressing PGM5-AS1 and sh-GDF10). All plasmids were purchased from Guangzhou RiboBio Co., Ltd. (Guangzhou, China). Cells were seeded into the 6-well plate in RPMI 1640 medium 24 h prior to transfection. When cell confluence reached about 80%, cells were resuspended in serum-free RPMI 1640 medium and then were seeded into the 6-well plate. Then, PCa cells were transfected using lipofectamine 2000 kit (Invitrogen, Carlsbad, CA, USA) for 6 h. Cells were cultured for 48 h in the renewed medium and collected for later use.

### RNA isolation and quantitation

Total RNA was extracted from tissues and cells using Trizol (Tel-Test. Austin, Texas, USA). RNA was reversely transcribed into cDNA and subjected to reverse transcription quantitative polymerase chain reaction (RT-qPCR) assay on the ABI7500 equipment (Applied Biosystems Inc., Foster City, CA, USA) using SYBR® Premix Ex Taq™ (Tli RNaseH Plus) kit (TaKaRa, Shiga, Japan). The expression of miR-587 was determined by means of TaqMan miRNA assay (Ambion, Austin, TX, USA) with U6 as the loading control. The expression of PGM5-AS1 and GDF10 were measured using PrimeScript RT-PCR kit (TaKaRa) with β-actin served as the internal reference of mRNAs. The primer sequences are shown in Table 1. Expression ratio of experimental gene to internal control was calculated based on the 2^−ΔΔCt^ method.

**Table 1:** Primer sequences for RT-qPCR

Gene	Forward sequence	Reverse sequence
PGM5-AS1	5′-GACTATGTTGTGAGCCTGCG-3′	5′-AAAAGGGGAGGGGCAATACA-3′
miR-587	5′-CCAGGCAAGAGAGAGTTGCTG-3′	5′-AGTCACAGGTGCAGACACATT-3′
GDF10	5′-GGACTTTGACGAGAAGACGATG-3′	5′-TCTTAGGCATGGGGAACTCAC-3′
U6	5′-CTCGCTTCGGCAGCACA-3′	5′-AACGCTTCACGAATTTGCGT-3′
β-actin	5′-CCTGGCACCCAGCACAAT-3′	5′-GCCGATCCACACGGAGTACT-3′

*RT-qPCR* reverse transcription quantitative polymerase chain reaction, *PGM5-AS1* homo sapiens PGM5 antisense RNA 1, *miR* microRNA, *GDF10* growth and differentiation factor 10

### Western blot analysis

The total protein was extracted from tissues after treatment of Radio Immunoprecipitation Assay lysis buffer (R0010, Solarbio, Shanghai, China) containing phenylmethylsulfonyl fluoride. The supernatant was collected following incubation for 30 min on ice and 4 min of centrifugation at 12,000 r/min. The protein concentration was quantified according to instructions in the bicinchoninic acid protein assay kit (23225, Pierce Biotechnology, IL, USA). Next, proteins were resolved on 10% sodium dodecyl sulfate polyacrylamide gel electrophoresis, electro-transferred to polyvinylidene fluoride membrane (blocked with 5% skim milk), and probed with rabbit antibodies to Ki67 (1:1000, ab16667, Abcam, Cambridge, UK), proliferating cell nuclear antigen (PCNA) (1:1000, ab18197, Abcam), cleaved caspase-3 (1:500, ab49822, Abcam), B-cell lymphoma-2 (Bcl-2) (1:1000, ab32124, Abcam), Bcl-2 associated protein X (Bax) (1:1000, ab32503, Abcam), receptor-interacting protein kinase3 (RIP3) (1:1000, ab222320, Abcam), cyclophilinA (1:1000, ab126738, Abcam), GDF10 (1:1000, ab235005, Abcam), and β-actin (1:1000, ab8224, Abcam). The membranes were incubated with secondary antibody of horseradish peroxidase-conjugated goat anti-rabbit immunoglobulin G (IgG) (1:5000, ab205718, Abcam) for 1 h and washed with Tris Buffered saline/Tween. Proteins were visualized with enhanced chemiluminescence and quantified with Quantity One software.

### Enzyme linked immunosorbent assay (ELISA)

A lactic acid dehydrogenase (LDH) ELISA kit (Nanjing Jiancheng Bioengineering Institute, Nanjing, Jiangsu, China) was used to detect cell necrosis with reference to the manual.

### 5-Ethynyl-2′-deoxyuridine (EdU) assay

After 48-h of transfection, PCa cells were incubated for 2 h in EdU medium with 100 μL per well, followed by incubation with 2 mg/mL glycine for 5 min. After treatment of phosphate buffer saline (PBS) containing 0.5% Triton X-100 as permeating agent for 10 min, cells were incubated in the 1 × Apollo staining solution in the dark for 30 min. Then, cells were immersed in 1 × Hoechst 33,342 reaction fluid for 30 min in the dark at room temperature. After staining, anti-fluorescence quenching agent was added to each well. Six to ten fields of view were randomly selected for each well for observation under a fluorescence microscope.

### Clonogenic assay

Cells were seeded onto a six-well plate (500 cells/well) and cultured for 14 days. The colonies were fixed in 10% methanol for 15 min and stained with 0.5% crystal violet for 30 min. Then the cells were photographed and counted.

### Flow cytometric analysis of apoptosis

Cells apoptosis was examined by flow cytometry with fluorescein isothiocyanate (FITC)-labeled Annexin V (Annexin V-FITC) and propidium iodide (PI) double staining (Sigma-Aldrich, St Louis, MO, USA). The cells were trypsinized and the number of sample cells was adjusted to 1 × 10^6^ cells/mL. Afterwards, cells were centrifuged to remove supernatant and fixed with 70% ethanol at 4 °C overnight. After another centrifugation, cells were resuspended in 200 μL binding buffer, and incubated with 10 μL Annexin V-FITC and 5 μL PI for 15 min at room temperature in the dark. Next, 300 μL binding buffer was added and cell apoptosis was examined using a FACS Calibur flow cytometer (Becton Dickinson, San Jose, CA, USA).

### *Fluorescence *in situ* hybridization (FISH) assay*

The subcellular localization of PGM5-AS1 in PCa cells was identified using FISH assay according to the protocols of lncRNA FISH Probe Mix (Red) (Guangzhou RiboBio Co., Ltd., Guangzhou, China). Cells were seeded into the 24-well plate (6 × 10^4^ cells/well). Cells at 80% confluence were fixed with 4% polyformaldehyde at room temperature and treated with protease K (2 μg/mL), glycine and ethylphthalide reagent. Subsequently, cells were cultured in 250 μL pre-hybridization solution at 42 °C for 1 h. Next, with pre-hybridization solution removed, cells were hybridized overnight at 42 °C in 250 μL hybridization solution supplemented with biotin-labeled antisense PGM5-AS1 probe (300 ng/mL, Shanghai Genechem Co., Ltd., China). Then cells were stained with 4′,6-diamidino-2-phenylindole diluted by PBS Tween-20 (1:800) for 5 min in a 24-well plate. Finally, cells sealed in antifade mounting medium were observed under a fluorescence microscope (Olympus, Tokyo, Japan) with five fields of vision randomly selected.

### Nude mice xenografted with PCa cells

Nude mice (4—6 weeks old, weight: 16–20 g, Laboratory Animal Center of Chinese Academy of Medical Sciences, Beijing, China) were assigned to five treatment groups: (1) blank (mice without any treatment), (2) oe-NC (mice injected with cells transfected with oe-NC), and (3) oe-PGM5-AS1 (mice injected with cells overexpressing PGM5-AS1), with 5 mice in each group. After 48-h of transfection, the concentration of PCa cells was adjusted to 4 × 10^5^ cells/mL. Then, a 0.5 mL volume of cell suspension was injected subcutaneously into the back of each nude mouse in accordance to establish PCa models in the nude mice. Tumor volume of was measured every 7 days and calculated according to the following formula: tumor volume = 0.5 × a × b^2^, where a represents the longest diameter and b represents the shortest diameter. The mice were euthanized at 28 day after xenografting, and the tumor tissues were removed and weighed. Half portions of each tumor were fixed with 4% methanol for immunohistochemical analysis and terminal deoxynucleotidyl transferase mediated 2′-deoxyuridine 5′-triphosphate nick-end labeling (TUNEL) assays, and the other half was stored at − 80 °C for molecular experiments.

### Immunohistochemistry

The removed tumor tissues of nude mice were embedded in paraffin, which were baked in a 60 °C oven for 1 h, sectioned, deparaffinized, and rehydrated in gradient ethanol. The activity of endogenous peroxidase was blocked by distilled water containing 0.3% hydrogen peroxide. Then, the sections were washed with TBS buffer saline (Dako, Glostrup, Denmark) and incubated overnight with primary anti-rabbit polyclonal antibody to Ki67 (ab16667, 1:1000, Abcam) at 4 °C. Next, the sections were incubated with horseradish peroxidase-labeled goat anti-rabbit IgG (1:1000, ab6721, Abcam) in a 37 °C water bath for 30 min. Then, sections were re-stained with hematoxylin for 2 min. Finally, a negative control was set for each antibody and each specimen.

### TUNEL assay

First, 4% methanol-fixed tumor tissues were sliced into 4 μm thick sections. A TUNEL kit (Boster Biological Technology Co., Ltd., Wuhan, Hubei, China) was utilized to evaluate the apoptosis of tumor tissues in accordance with the manufacturer’s protocols.

### Dual-luciferase reporter gene assay

Target genes of miR-587 were predicted using the publicly available microRNA.org website for biological prediction. Artificially synthesized GDF10 3′untranslated region (3′UTR) gene fragments were introduced into pMIR-reporter (Huayueyang Biotechnology Co., Ltd., Beijing, China) using endonuclease sites SpeI and Hind III. Complementary sequence mutation sites of seed sequences were designed on the wild type (WT) of GDF10. The target fragments were inserted into the pMIR-reporter plasmid after restriction endonuclease digestion by T4 DNA ligase. The correctly sequenced luciferase reporter plasmids WT and mutant (MUT) were co-transfected into HEK-293 T cells (Shanghai Beino Biotechnology Co., Ltd., China) with miR-587 mimic, respectively. Cells were collected and lysed 48 h after transfection. Luciferase activity was examined using Glomax 20/20 Luminometer (Promega Corp., WI, USA) and a dual Luciferase detection kit (K801-200, BioVision, Palo Alto, USA). The binding relationship between PGM5-AS1 and miR-587 was examined by the same method.

### RNA-binding protein immunoprecipitation (RIP)

PCa cells were washed with precooled PBS and trypsinized. Then, the cells were lysed in a buffer containing RNase (Gibco Life Technologies) and protease inhibitor cocktail (Roche, Basel, Switzerland). The lysate was centrifuged (1200×*g*) for 30 min to obtain the supernatant. Next, the protein G agarose beads, Argonaute 2 (Ago2) antibody (P10502500, Otwo Biotech Inc., Shenzhen, China) and IgG (Sigma-Aldrich) were incubated at 4 °C for 2 h and then were added into the supernatant for further incubation overnight at 4 °C. The beads were eluted three times, and RNA was extracted from magnetic beads by addition of Trizol Reagent (Invitrogen). Expression of PGM5-AS1 and miR-587 was examined by RT-qPCR.

### RNA pull-down

PCa cells were transfected with 50 nM biotin-labeled bio-miR-587, bio-miR-587-mut and corresponding NC-bio for 48 h. Cells were then incubated in specific lysis buffer (Ambion, Austin, Texas, USA) for 10 min and then the mixture were centrifuged (14,000×*g*) to obtain the supernatant. Protein lysate was incubated with M-280 streptavidin beads (S3762, Sigma-Aldrich) which were pre-coated with RNase-free bovine serum albumin and yeast tRNA (TRNABAK-RO, Sigma-Aldrich). Next, the beads were incubated at 4 °C for 3 h. The binding RNA was purified by the Trizol method, and the PGM5-AS1 expression was examined by RT-qPCR.

### Statistical analysis

Statistical analyses were conducted by SPSS 21.0 (IBM-SPSS Inc., Armonk, NY, USA). Measurement data are summarized as mean ± standard deviation. The data were confirmed with normal distribution and homogeneity of variance after tests. Comparison between two groups was conducted by independent sample *t*-test. Comparisons among multiple groups were conducted by one-way analysis of variance (ANOVA) with Tukey’s post hoc test. Statistical analysis in relation to time-based measurements within each group was realized using repeated measures ANOVA, followed by a Bonferroni’s *post-hoc* test. A value of *p* < 0.05 indicates significant difference.

## Results

### PGM5-AS1 is poorly expressed in PCa cell line

**Fig. 1:**
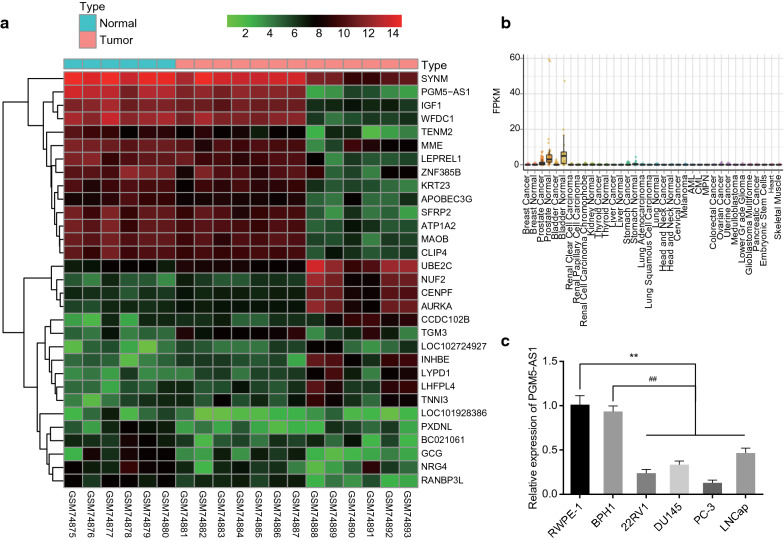
Poorly expressed PGM5-AS1 is observed in PCa cell lines. **a** The graphical heatmap (GSE3325) for differentially expressed lncRNAs. The X axis shows sample numbers, and the Y axis shows differentially expressed lncRNAs. In each panel, the expression level is depicted according to the color score shown at the upper right. **b** The expression of PGM5-AS1 in PCa in MiTranscriptome database. **c** The relative expression of PGM5-AS1 in each cell line as measured by RT-qPCR. *p < 0.05, **p < 0.01 vs. the RWPE-1 cell line. ^#^*p* < 0.05, ^##^*p* < 0.01 vs. the BPH1 cell line. The measurement data were summarized as mean ± standard error and analyzed using one-way ANOVA, followed by Tukey's post hoc test. The experiment was repeated three times independently

Based on the analysis of GSE3325 microarray dataset (Fig. 1a), 11 genes including UBE2C, NUF2, CENPF, AURKA, CCDC102B, TGM3, LOC102724927, INHBE, LYPD1, LHFPL4, TNNI3, were highly expressed in PCa samples, whereas 20 genes (SYNM, PGM5-AS1, IGF1, WFDC1, TENM2, MME, LEPREL1, ZNF385B, KRT23, APOBEC3G, SFRP2, ATP1A2, MAOB, CLIP4, LOC101928386, PXDNL, BC021061, GCG, NRG4, RANBP3L) were poorly expressed in PCa samples compared with normal samples. Combined with the screening through MiTranscriptome database (https://mitranscriptome.org) (Fig. 1b), we find that the expression of PGM5-AS1 in patients with PCa is downregulated. Consistently, RT-qPCR assays indicated that the four PCa cell lines (PC-3, 22RV1, DU145 and LNCap) showed lower PGM5-AS1 expression than the two normal prostate epithelial cell lines (BPH1 and RWPE-1) (*p* < 0.05). The expression level of PGM5-AS1 in each cell line was ranked in ascending order as follows: PC-3 < 22RV1 < DU145 < LNCap < BPH1 < RWPE-1 (Fig. 1c). In addition, PC-3 cells are capable of growing androgen-independently, proliferating and forming distant metastatic lesions in animals, thus modelling closely the clinical pathophysiology of castration resistant PCa [14]. Therefore, we selected the PC-3 cell line for subsequent experiments.

### PGM5-AS1 overexpression suppresses proliferation, colony formation and enhances apoptosis of PCa cells

**Fig. 2:**
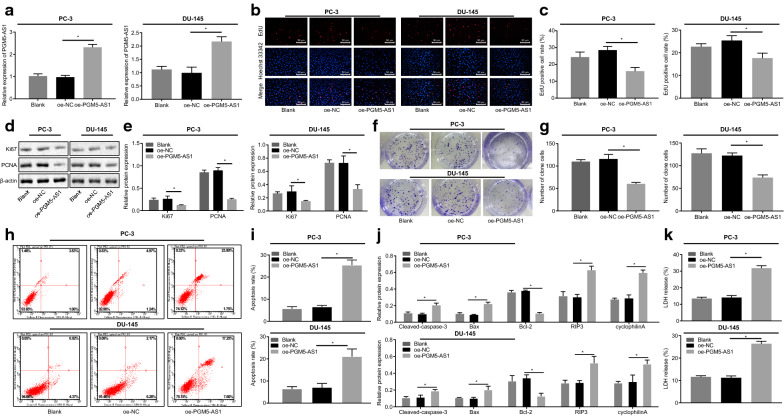
PGM5-AS1 overexpression inhibits the proliferation and colony formation of PC-3 and DU-145 cells and promotes their apoptosis. **a** The relative expression of PGM5-AS1 in PC-3 and DU-145 cells after transfection through RT-qPCR assay. **b** The representative images of cell proliferation in transfected PC-3 cells as measured by EdU assay (×200). **c** The quantitative analysis of cell proliferation in transfected PC-3 cells as measured by EdU assay. **d** The protein bands of expression of Ki67 and PCNA in transfected PC-3 cells through Western blot analysis. **e** The quantitative analysis of expression of Ki67 and PCNA in transfected PC-3 cells through Western blot analysis. **f** The representative images of cell colony formation in transfected PC-3 cells. **g** The quantitative analysis of cell colony formation in transfected PC-3 cells. **h** The flow cytometric detection of cell apoptosis in transfected PC-3 cells. **i** The quantitative analysis of cell apoptosis in transfected PC-3 cells detected by flow cytometric analysis. **j** The quantitative analysis of expression of cleaved caspase-3, Bax, RIP3, cyclophilinA and Bcl-2 in transfected PC-3 cells through Western blot analysis. **k** LDH release determined by ELISA. *p < 0.05 vs. the oe-NC group (PCa cells treated with oe-NC). The measurement data were summarized as mean ± standard error and analyzed using one-way ANOVA, followed by Tukey's post hoc test. The experiment was repeated three times independently

After transfection, the PGM5-AS1 expression was further determined in PC-3 and DU-145 cells. No obvious difference was observed in PGM5-AS1 expression between untreated cells and those treated with oe-NC (*p* > 0.05), but the expression of PGM5-AS1 was increased in the cells transduced with PGM5-AS1 overexpression plasmid versus those treated with oe-NC (*p* < 0.05) (Fig. 2a). These results confirmed successful transfection of PGM5-AS1 in PCa cells. EdU proliferation assays identified reduced proliferation ability in the cells overexpressing PGM5-AS1 versus those treated with oe-NC (*p* < 0.05), and no significant difference between untreated cells and those treated with oe-NC (*p* > 0.05) (Fig. 2b, c). Western blot analysis showed significantly lower expression of Ki67 and PCNA in the cells overexpressing PGM5-AS1 versus those treated with oe-NC (*p* < 0.05), and no obvious difference between the untreated cells and those treated with oe-NC (*p* > 0.05) (Fig. 2d, e). Moreover, based on experimental data from the clonogenic assay (Fig. 2f, g) and flow cytometry (Fig. 2h, i), cells overexpressing PGM5-AS1 exhibited diminished cell colony formation and elevated cell apoptosis rate as compared with untreated cells (*p* < 0.05). Consistent with this, Western blot analysis also revealed elevated expression of pro-apoptotic proteins (cleaved caspase-3, Bax, RIP3 and cyclophilinA) and reduced anti-apoptotic Bcl-2 (*p* < 0.05) (Fig. 2j). Further ELISA analysis showed that LDH release was promoted by oe-PGM5-AS1 (*p* < 0.05) (Fig. 2k). Also, we observed no appreciable difference in LDH release between untreated cells and those treated with oe-NC (*p* > 0.05). All these results indicate that PGM5-AS1 overexpression attenuated proliferation, colony formation and enhanced apoptosis of PCa cells.

### PGM5-AS1 competitively binds to miR-587

**Fig. 3:**
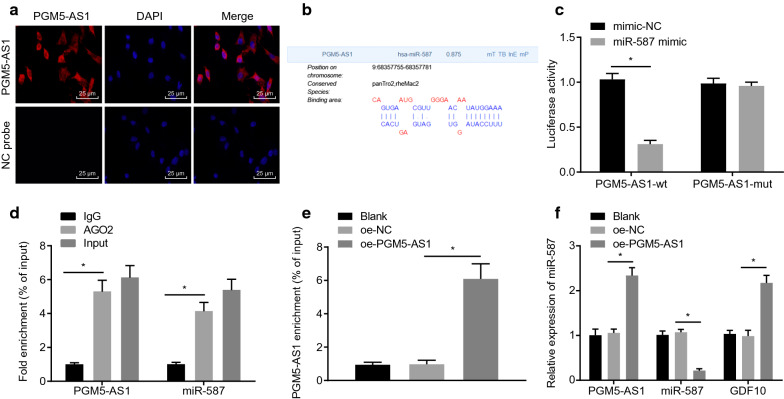
PGM5-AS1 competitively binds to miR-587. **a** The subcellular localization of PGM5-AS1 as evaluated by RNA-FISH (×400). **b** miR-587 that binds to PGM5-AS1 was screened out by DIANA Tools database. **c** The verification of the binding relationship between PGM5-AS1 and miR-587 through dual-luciferase activity detection. *p < 0.05 vs. the mimic-NC group. **d** RIP assay to verify the binding relationship between PGM5-AS1 and miR-587. *p < 0.05 vs. the IgG group. **e** RNA pull-down analysis of the binding relationship between PGM5-AS1 and miR-587. *p < 0.05 vs. the NC-bio group. **f** miR-587, PGM5-AS1 and GDF10 expression in PGM5-AS1 overexpressing cells measured by means of RT-qPCR assay. The measurement data are summarized as mean ± standard error. Comparison between two groups was conducted by independent sample *t*-test. Comparisons among multiple groups were conducted by one-way ANOVA with Tukey’s post hoc test. The experiment was repeated three times independently

RNA-FISH analysis showed that PGM5-AS1 expression was mainly localized in cytoplasm (Fig. 3a). With the assistance of the DIANA Tools database (Tools https://diana.imis.athena-innovation.gr/DianaTools/index.php?R=site/page&view=software), we screened out miR-587 (score = 0.875) as the mRNA with the greatest likelihood of binding to PGM5-AS1 (Fig. 3b). Dual-luciferase assay demonstrated that, relative to cells transfected with miR-587 mimic-NC + PGM5-AS1-WT, the luciferase activity was lower in cells transfected with miR-587 mimic + PGM5-AS1-WT (*p* < 0.05), while no obvious difference was observed in the cells transfected with miR-587 mimic + PGM5-AS1-MUT (*p* > 0.05), thus suggesting a binding relationship between PGM5-AS1 and miR-587 (Fig. 3c). In addition, in the RIP assay, expression of PGM5-AS1 and miR-587 was diminished in the presence of Ago2/IgG relative to the input (*p* < 0.05) (Fig. 3d). In the RNA pull-down experiment, PGM5-AS1 expression was significantly elevated in the miR-587-bio group when compared with that in the NC-bio group, suggesting that PGM5-AS1 was indeed enriched in samples pulled down by miR-587 probes (Fig. 3e). RT-qPCR assay (Fig. 3f) identified elevated PGM5-AS1 expression and reduced miR-587 and GDF10 expression in cells overexpressing PGM5-AS1 versus those treated with oe-NC (*p* < 0.05). No significant difference was witnessed in miR-587, PGM5-AS1 or GDF10 expression between untreated cells and those treated with oe-NC (*p* > 0.05). These findings indicate that PGM5-AS1 binds to miR-587.

### miR-587 upregulation enhances proliferation, colony formation and represses apoptosis of PC-3 cell line by targeting GDF10

**Fig. 4:**
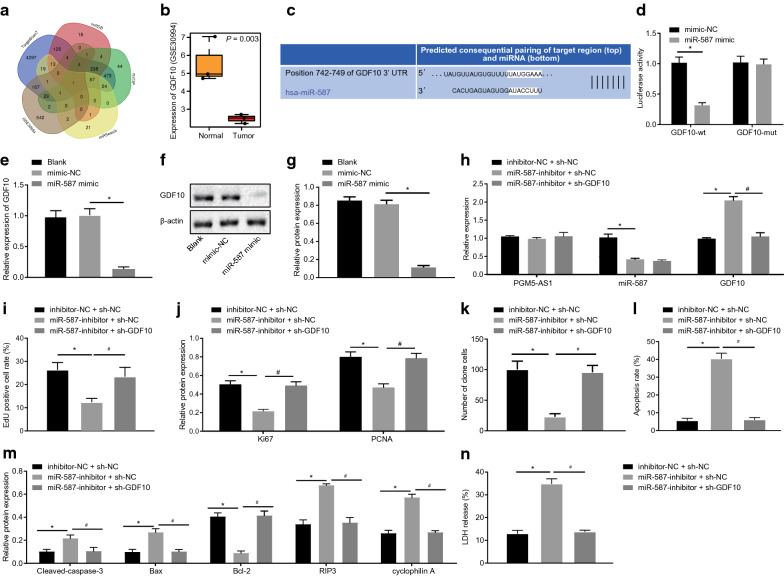
miR-587 upregulation accelerates proliferation, colony formation and impedes apoptosis of PC-3 cell line by targeting GDF10. **a** The prediction of target genes of miR-587. **b** GDF10 expression in GSE30994 dataset. **c** The predictive analysis on online website (https://www.targetscan.org/) for binding sites between miR-587 and GDF10. **d** The dual-luciferase reporter assay to verify miR-587 binding to GDF10. **e** RT-qPCR detection of relative expression of GDF10, PGM5-AS1 and miR-587 in PCa cells treated with miR-587 mimic. **f** The protein bands of expression of GDF10 protein in PCa cells treated with miR-587 mimic through Western blot analysis. **g** The quantitative analysis of expression of GDF10 protein in PCa cells treated with miR-587 mimic through Western blot analysis. **h** The relative expression of miR-587, GDF10 and PGM5-AS1 in transfected PCa cells as evaluated by RT-qPCR; *p < 0.05 vs. the blank group (untreated PCa cells). **i** The quantitative analysis of cell proliferation in transfected PC-3 cells as measured by EdU assay. **j** The protein expressions of Ki67 and PCNA in transfected PC-3 cells through Western blot analysis. **k** The quantitative analysis of cell colony formation in transfected PC-3 cells. **l** The quantitative analysis of cell apoptosis in transfected PC-3 cells detected by flow cytometric analysis. **m** The quantitative analysis of expression of cleaved caspase-3, Bax, RIP3, cyclophilinA and Bcl-2 in transfected PC-3 cells through Western blot analysis. **n** LDH release determined by ELISA. *p < 0.05 vs. the mimic-NC group (PCa cells treated with mimic-NC) or the inhibitor-NC + sh-NC group (PCa cells treated with inhibitor-NC + sh-NC). ^#^*p* < 0.05 vs. the miR-587 inhibitor + sh-NC group (PCa cells treated with miR-587 inhibitor + sh-NC). The measurement data are summarized as mean ± standard error. Comparison between two groups was conducted by independent sample *t*-test. Comparisons among multiple groups were conducted by one-way ANOVA with Tukey’s post hoc test. The experiment was repeated three times independently

The target gene GDF10 of miR-587 was screened out by intersecting the microSearch V3.0 (https://www.exiqon.com/miRSearch), microDB (https://www.mirdb.org/) and mirDIP (https://ophid.utoronto.ca/mirDIP/) databases with the poorly-expressed genes in the GSE30994 dataset (Fig. 4a, b). Predictive analysis of the TargetScan database (https://www.TargetScan.org/) suggested binding sites between miR-587 and GDF10 3′UTR (Fig. 4c). Moreover, data obtained from dual-luciferase assays verified that, compared with mimic-NC group, the luciferase activity was decreased in the miR-587 mimic and GDF10-WT group (*p* < 0.05), while no obvious difference was observed in the miR-587 mimic group and GDF10-MUT group (*p* > 0.05) (Fig. 4d). Subsequent RT-qPCR and Western blot assays confirmed that the treatment with miR-587 mimic led to a decline in GDF10 expression versus cells treated with mimic-NC (*p* < 0.05) (Fig. 4e–g). The transfection efficiency was evaluated as shown in Fig. 4h, which suggested that the transfected cells could be used for subsequent experiments. EdU proliferation assay indicated suppressed cell proliferation ability in the presence of miR-587 inhibitor + sh-NC versus the treatment of inhibitor-NC + sh-NC (*p* < 0.05) (Fig. 4i), which was consistent with diminished expression of Ki67 and PCNA as measured by Western blot assay (*p* < 0.05) (Fig. 4j). Additionally, cell proliferation ability and expression of Ki67 and PCNA were both elevated in response to miR-587 inhibitor + sh-GDF10 versus miR-587 inhibitor + sh-NC group (*p* < 0.05). Moreover, based on experimental data from the clonogenic assay (Fig. 4k), cell colonies were diminished upon treatment of miR-587 inhibitor + sh-NC versus the treatment with inhibitor-NC + sh-NC (*p* < 0.05), but were elevated in response to miR-587 inhibitor + sh-GDF10 relative to miR-587 inhibitor + sh-NC (*p* < 0.05). In addition, cell apoptosis as evaluated by flow cytometry (Fig. 4l) was enhanced upon treatment with miR-587 inhibitor + sh-NC versus inhibitor-NC + sh-NC (*p* < 0.05), which concurred with findings of increased pro-apoptotic proteins (cleaved caspase-3, Bax, RIP3 and cyclophilinA) and reduced anti-apoptotic Bcl-2 (*p* < 0.05) (Fig. 4m). The cell apoptosis was repressed upon treatment of miR-587 inhibitor + sh-GDF10 versus treatment of miR-587 inhibitor + sh-NC (*p* < 0.05), concurring with diminished pro-apoptotic proteins (cleaved caspase-3, Bax, RIP3 and cyclophilinA) and elevated anti-apoptotic Bcl-2 protein levels (*p* < 0.05). ELISA revealed significantly increased LDH release in cells treated with miR-587-inhibitor + sh-NC, while further addition of sh-GDF10 decreased LDH release (*p* < 0.05) (Fig. 4n). All these results indicated that miR-587 upregulation facilitated proliferation, colony formation and impeded apoptosis of PC-3 cell line by targeting GDF10.

### *PGM5-AS1 inhibits malignant progression of PCa cells *via* upregulation of GDF10 by binding to miR-587*

**Fig. 5:**
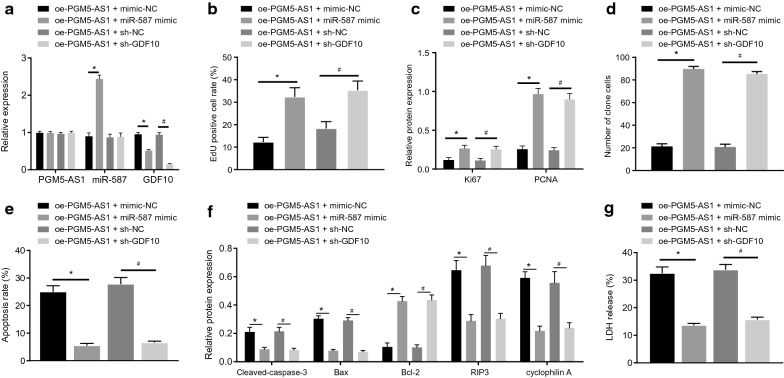
PGM5-AS1 inhibits malignant progression of PCa cells via upregulation of GDF10 by binding to miR-587. **a** The relative expression of PGM5-AS1, miR-587 and GDF10 in transfected PCa cells as evaluated by RT-qPCR; *p < 0.05 vs. the blank group (untreated PCa cells). **b** The quantitative analysis of cell proliferation in transfected PC-3 cells as measured by EdU assay. **c** The quantitative analysis of expression of Ki67 and PCNA in transfected PC-3 cells through Western blot analysis. **d** The quantitative analysis of cell colony formation in transfected PC-3 cells. **e** The quantitative analysis of cell apoptosis in transfected PC-3 cells detected by flow cytometric analysis. **f** The protein bands of expression of cleaved caspase-3, Bax, RIP3, cyclophilinA and Bcl-2 in transfected PC-3 cells through Western blot analysis. **g** LDH release determined by ELISA. *p < 0.05 vs. the oe-PGM5-AS1 + mimic-NC group (PCa cells overexpressing PGM5-AS1 treated with mimic-NC). ^#^*p* < 0.05 vs. the oe-PGM5-AS1 + sh-NC group (PCa cells overexpressing PGM5-AS1 treated with sh-NC). The measurement data are summarized as mean ± standard error. Comparison between two groups was conducted by independent sample *t*-test. The experiment was repeated three times independently

The transfection efficiency of PCa cells was evaluated by RT-qPCR (Fig. 5a) to validate subsequent experiments. EdU proliferation assay showed enhanced cell proliferation ability in the presence of miR-587 mimic in cells overexpressing PGM5-AS1 versus the treatment with mimic-NC (*p* < 0.05) (Fig. 5b), which was consistent with elevated expression of Ki67 and PCNA as measured by Western blot assay (*p* < 0.05) (Fig. 5c). Moreover, in response to sh-GDF10, cell proliferation ability and expression of Ki67 and PCNA were both repressed in cells overexpressing PGM5-AS1 versus the treatment with sh-NC (*p* < 0.05). Moreover, based on experimental data of clonogenic assays (Fig. 5d), cell colonies numbers were elevated upon treatment of miR-587 mimic in cells overexpressing PGM5-AS1 versus the treatment with mimic-NC (*p* < 0.05), but colony numbers were diminished in response to sh-GDF10, relative to sh-NC treatment (*p* < 0.05). In addition, cell apoptosis as evaluated by flow cytometry (Fig. 5e) was suppressed upon treatment of miR-587 mimic in cells overexpressing PGM5-AS1 versus the treatment with mimic-NC (*p* < 0.05), corresponding to diminished pro-apoptotic proteins (cleaved caspase-3, Bax, RIP3 and cyclophilinA) and elevated anti-apoptotic Bcl-2 protein (*p* < 0.05) (Fig. 5f). The cell apoptosis was facilitated upon treatment of sh-GDF10 in cells overexpressing PGM5-AS1 versus treatment with sh-NC (*p* < 0.05), corresponding to upregulated pro-apoptotic proteins (cleaved caspase-3, Bax, RIP3 and cyclophilinA) and decreased anti-apoptotic Bcl-2 protein (*p* < 0.05). ELISA showed that, compared with oe-PGM5-AS1 treatment alone, LDH release was increased in response to treatment of oe-PGM5-AS1 + miR-587 mimic yet decreased in response to treatment with oe-PGM5-AS1 + sh-GDF10 (*p* < 0.05) (Fig. 5g). In addition, similar results were observed in another PCa cell line DU-145 (Supplementary Fig. 1). The above results indicated that PGM5-AS1 upregulated GDF10 gene expression by binding to miR-587, resulting in inhibited PCa cell proliferation and colony formation, as well as promoted PCa cell apoptosis.

### PGM5-AS1 overexpression impedes tumor growth in nude mice

**Fig. 6:**
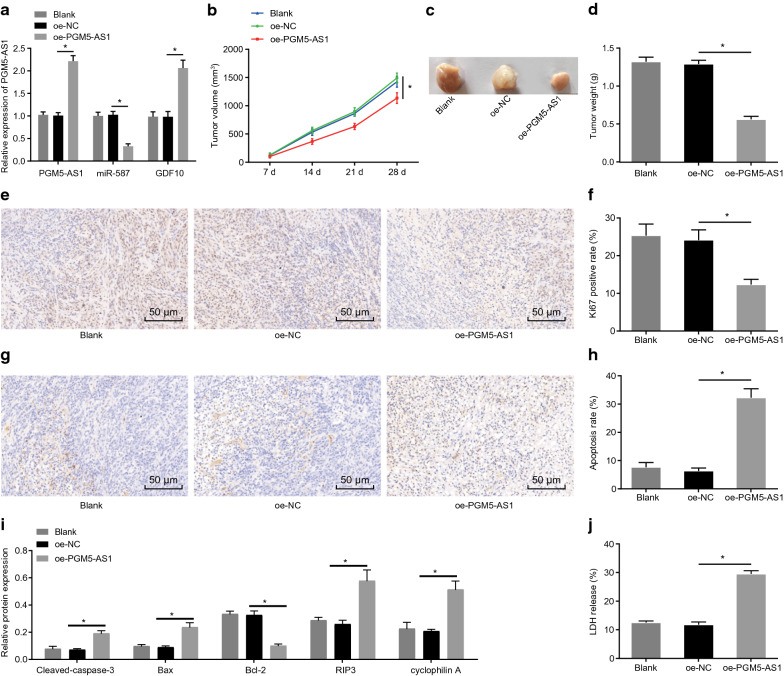
PGM5-AS1 overexpression suppresses prostatic tumorigenesis in nude mice. **a** The relative expression of PGM5-AS1, miR-587 and GDF10 in the xenograft prostatic tumor after transduction through RT-qPCR assay. **b** The quantitative analysis for volume change of the xenograft prostatic tumors in nude mice measured by a vernier caliper. **c** The representative images of the xenograft prostatic tumor in nude mice. **d** The quantitative analysis for weight of the xenograft prostatic tumor in nude mice. **e** The immunohistochemical staining of Ki67 expression in tumor tissues (×200). **f** The quantitative analysis of Ki67 expression in tumor tissues. **g** TUNEL staining of cell apoptosis of tumor tissues (×200). **h** The quantitative analysis of cell apoptosis of tumor tissues. **i** The protein bands of expression of cleaved caspase-3, Bax and Bcl-2 in tumor tissues through Western blot analysis. **j** LDH release determined by ELISA. n = 5. *p < 0.05 vs. the oe-NC group (nude mice injected with cells treated with oe-NC). The measurement data were summarized as mean ± standard deviation. Comparisons among multiple groups were conducted by one-way ANOVA with Tukey’s post hoc test. Statistical analysis in relation to time-based measurements within each group was realized using repeated measures ANOVA, followed by a Bonferroni’s *post-hoc* test. The experiment was repeated three times independently

Next, we proceeded to examine the expression and effect of PGM5-AS1 in vivo. PGM5-AS1 overexpression was successfully transduced into the tumors of nude mice, according to the downregulated miR-587 expression and upregulated PGM5-AS1 and GDF10 expression observed in tumor tissues from nude mice injected with cells transduced with PGM5-AS1 overexpression plasmid (Fig. 6a). In addition, we saw no obvious difference in PGM5-AS1, miR-587 or GDF10 expression in tumor tissues from untreated mice compared with those treated with oe-NC (*p* > 0.05) (Fig. 6a), and likewise no appreciable difference in tumor size and weight (*p* > 0.05) (Fig. 6b–d). However, we saw a significant reduction in size of the tumors overexpressing PGM5-AS1 versus those treated with oe-NC (*p* < 0.05) (Fig. 6b–d). Based on the obtained data of immunohistochemical (Fig. 6e, f) and TUNEL (Fig. 6g, h) assay, the tumors overexpressing PGM5-AS1 exhibited diminished Ki67 expression and enhanced apoptosis compared to those treated with oe-NC (*p* < 0.05). In addition, untreated mice and those treated with oe-NC also showed no remarkable differences in these markers (*p* > 0.05). Furthermore, Western blot analysis revealed elevated expression of pro-apoptotic proteins (cleaved caspase-3 and Bax) and reduced anti-apoptotic Bcl-2 protein (*p* < 0.05) (Fig. 6i, j). Also, there was no appreciable difference in these markers between untreated cells and those treated with oe-NC (*p* > 0.05). ELISA revealed significantly elevated LDH release in the presence of oe-PGM5-AS1 (Fig. 6k). All these results indicated that PGM5-AS1 overexpression suppressed the growth of xenograft prostatic tumor via the miR-587/GDF10 axis.

## Discussion

**Fig. 7:**
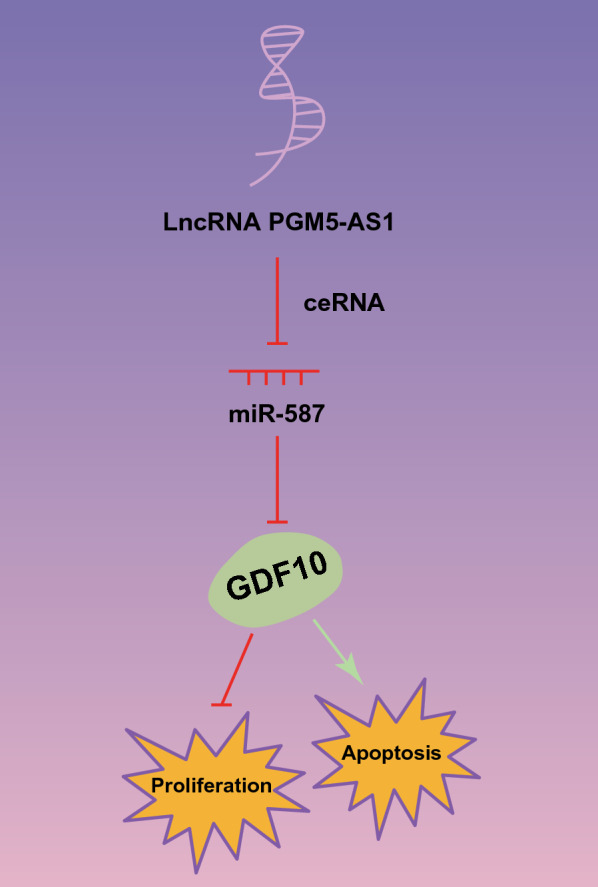
A graphic abstract showing PGM5-AS1 upregulates GDF10 expression by binding to miR-587, which leads to inhibited PCa cell proliferation, and promoted PCa cell apoptosis

PCa is widely recognized as a significant health problem affecting aging men [15]. Interestingly, the molecular mechanisms of PCa are related to the dysregulation of mRNAs, miRNAs or lncRNAs, which exert important effects on different biological processes related to cancer pathogenesis [8]. Aberrantly expressed lncRNAs may result in antineoplastic or tumorigenic functions by mediating carcinogenesis-related miRNAs or mRNAs [16]. In the current study, we found that PGM5-AS1 upregulated the GDF10 expression by sequestering miR-587, thus restricting proliferation and facilitating apoptosis of PCa cells (Fig. 7).

Present experimental data revealed low expression of PGM5-AS1 in PCa cell lines. Moreover, in vitro and in vivo experiments indicated that the ectopic expression of PGM5-AS1 could enhance PCa cell apoptosis and lower the proliferation and colony formation of PCa cells, as well as reducing prostatic tumorigenesis in xenografted nude mice. Extensive studies have documented the diagnostic and prognostic functions of lncRNAs in PCa, which may be abnormally upregulated as oncogenes (SNHG12) [17], or downregulated as tumor suppressors (BRE-AS1) [18]. A previously reported study highlighted the aberrantly low expression of PGM5-AS1 in colorectal cancer tissues and cells, and the anti-cancer potential of PGM5-AS1 overexpression in colorectal cancer [9]. In addition, results of functional assays in esophageal squamous cell carcinoma showed that ectopic expression of PGM5-AS1 restricted the malignant phenotype progression through impairment of the miR-466-mediated PTEN inhibition [10]. The tumor suppressive role of PGM5-AS1 having been revealed in PCa, we proceeded to seek the underlying functional mechanisms.

Recent evidence has deciphered that lncRNAs can bind to and sequester miRNAs as competitive endogenous RNAs, thereby curbing the direct effects of miRNA on downstream mRNAs [19]. After verification with dual-luciferase reporter, RIP and RNA pull down assays, we confirmed that PGM5-AS1 competitively bound to and inversely regulated miR-587. miR-587 has been previously elucidated to be a regulator of cell cycle progression via the TGF-β-SMAD signaling pathway [20]. The oncomiR property of miR-587 has been documented in the study of Zhang et al*.,* which demonstrated that inhibition of miR-587 could restore the 5-fluorouracil-induced cell apoptosis in colorectal cancer and even cause significant declines in drug resistance [11]. Largely in agreement with our present findings, upregulation of miR-587 has been identified in cervical cancer and specific inhibitor-induced miR-587 knockdown exerts inhibitory effects on tumor growth [21]. The rescue experiments in this study unearthed that miR-587 upregulation-induced by mimics could reverse the suppressed cell proliferation and colony formation triggered by PGM5-AS1 overexpression.

Next, we went on to seek the downstream target gene of miR-587 and to validate the above-mentioned findings. Results suggested that miR-587 targeted and negatively regulated the GDF10 gene, and more importantly, that miR-587 upregulation accelerated proliferatio and, colony formation, and diminished apoptosis of PCa cells by targeting GDF10. The function of miR-587 in cancer is presumably mediated by its regulation of target genes. For instance, a prior study has demonstrated that miR-587 could target and negatively regulate the target gene PTEN, by which mechanism miR-587 present a promising diagnostic marker and risk factor for metabolic syndrome [22]. Multiple studies have suggested that GDF10, a member of the TGF-β family, was downregulated in various tumors. For example, the study of Dai et al*.* delineated that GDF10 expression is suppressed in lung cancer, and that the aberrant downregulation of GDF10 contributes to the tumor growth in the lung [23]. GDF10 was also reported to function as a tumor suppressor in epithelial cells of breast cancer and to restrict their proliferation and epithelial-mesenchymal transition [24]. Molina et al*.* focused on breast cancer, finding consistent results that GDF10 hypermethylation is a common epigenetic event in breast cancer, which leads to aberrant repression of GDF10 expression. Functional experiments in oral squamous cell carcinoma unraveled that overexpression of GDF10, triggered by type III TGF-β receptor via the TGF-β-SMAD2/3 signaling pathway, resulted in appreciable inhibition in cell proliferation, migration and invasion [25]. Additionally, a previous study deciphered that PGM5-AS1, which is downregulated in colorectal cancer, functioned as a molecular sponge to mediate SMAD4 expression by sponging miR-100-5p [26]. In our study, PGM5-AS1 abrogated malignant progression of PCa Cells via upregulation of GDF10 by sequestering miR-587 (Additional file 1: Fig. S1).

## Conclusions

We conclude that the PGM5-AS1/miR-587/GDF10 axis shed new light for understanding the aggressive potential of PCa, and for the development of better diagnostic methods and therapies. PGM5-AS1 upregulates GDF10 expression by binding to miR-587, which leads to suppressed PCa cell proliferation, and promoted PCa cell apoptosis. However, we need to confirm the present preclinical results in larger patient cohorts and identify the best strategy to upregulate or downregulate PGM5-AS1 so as to limit any adverse effects. Most importantly, we need to obtain a sufficient number of clinical samples to support a receiver operator characteristic curve for further application of present findings in the clinic.

## Supplementary information

**Additional file 1: Fig. S1.** PGM5-AS1 inhibits malignant progression of PCa cells via upregulation of GDF10 by binding to miR-587. **A** The relative expression of PGM5-AS1, miR-587 and GDF10 in transfected DU-145 cells as evaluated by RT-qPCR. **B** The representative images of EDU staining of transfected DU-145 cells. **C** The quantitative analysis of cell proliferation in transfected DU-145 cells as measured by EdU assay. **D** The protein expression of Ki67 and PCNA in transfected DU-145 cells through Western blot analysis. **E** The representative images of cell colony formation in transfected DU-145 cells. **F** The quantitative analysisof cell colony formation in transfected DU-145 cells. **G** The representative images of cell apoptosis in transfected DU-145 cells detected by flow cytometric analysis. **H** The quantitative analysis of cell apoptosis in transfected DU-145 cells detected by flow cytometric analysis. **I** The protein expression of cleaved caspase-3, Bax, RIP3, cyclophilinA and Bcl-2 in transfected DU-145 cells through Western blot analysis. **J**LDH release in transfected DU-145 cells determined by ELISA. * *p* < 0.05 vs. the oe-PGM5-AS1 + mimic-NC group (DU-145 cells overexpressing PGM5-AS1 treated with mimic-NC). # *p* < 0.05 vs. the oe-PGM5-AS1 + sh-NC group (DU-145 cells overexpressing PGM5-AS1 treated with sh-NC). The measurement data are summarized as mean ± standard error. Comparison between two groups was conducted by independent sample *t*-test. The experiment was repeated three times independently.

## Data Availability

The datasets generated/analyzed during the current study are available.

## Abbreviations

PCa: Prostate cancer
PSA: Prostate-specific antigen
GEO: Gene Expression Omnibus
DEGs: Differentially expressed genes
FISH: Fluorescence in situ hybridization
WT: Wild type

## References

[1] Siegel RL, Miller KD, Jemal A (2019). Cancer statistics, 2019. CA Cancer J Clin.

[2] Curry SJ, Krist AH, Owens DK (2019). Annual report to the nation on the status of cancer, part II: Recent changes in prostate cancer trends and disease characteristics. Cancer.

[3] Chou R, Croswell JM, Dana T, Bougatsos C, Blazina I, Fu R, Gleitsmann K, Koenig HC, Lam C, Maltz A (2011). Screening for prostate cancer: a review of the evidence for the US Preventive Services Task Force. Ann Intern Med..

[4] Fenton JJ, Weyrich MS, Durbin S, Liu Y, Bang H, Melnikow J (2018). Prostate-specific antigen-based screening for prostate cancer: evidence report and systematic review for the US preventive services task force. JAMA.

[5] Miller KD, Siegel RL, Lin CC, Mariotto AB, Kramer JL, Rowland JH, Stein KD, Alteri R, Jemal A (2016). Cancer treatment and survivorship statistics, 2016. CA Cancer J Clin.

[6] Wesselhoeft RA, Kowalski PS, Parker-Hale FC, Huang Y, Bisaria N, Anderson DG (2019). RNA circularization diminishes immunogenicity and can extend translation duration in vivo. Mol Cell.

[7] Xu YH, Deng JL, Wang G, Zhu YS (2019). Long non-coding RNAs in prostate cancer: functional roles and clinical implications. Cancer Lett.

[8] Ye Y, Li SL, Wang SY (2018). Construction and analysis of mRNA, miRNA, lncRNA, and TF regulatory networks reveal the key genes associated with prostate cancer. PLoS ONE.

[9] Zhang Q, Ding Z, Wan L, Tong W, Mao J, Li L, Hu J, Yang M, Liu B, Qian X (2019). Comprehensive analysis of the long noncoding RNA expression profile and construction of the lncRNA-mRNA co-expression network in colorectal cancer. Cancer Biol Ther..

[10] Zhihua Z, Weiwei W, Lihua N, Jianying Z, Jiang G (2019). p53-induced long non-coding RNA PGM5-AS1 inhibits the progression of esophageal squamous cell carcinoma through regulating miR-466/PTEN axis. IUBMB Life.

[11] Zhang Y, Talmon G, Wang J (2015). MicroRNA-587 antagonizes 5-FU-induced apoptosis and confers drug resistance by regulating PPP2R1B expression in colorectal cancer. Cell Death Dis.

[12] Zhao R, Lawler AM, Lee SJ (1999). Characterization of GDF-10 expression patterns and null mice. Dev Biol.

[13] Tandon M, Gokul K, Ali SA, Chen Z, Lian J, Stein GS, Pratap J (2012). Runx2 mediates epigenetic silencing of the bone morphogenetic protein-3B (BMP-3B/GDF10) in lung cancer cells. Mol Cancer.

[14] Zhang S, Wang Y, Chen Z, Kim S, Iqbal S, Chi A, Ritenour C, Wang YA, Kucuk O, Wu D (2013). Genistein enhances the efficacy of cabazitaxel chemotherapy in metastatic castration-resistant prostate cancer cells. Prostate.

[15] Qaseem A, Barry MJ, Denberg TD, Owens DK, Shekelle P (2013). Screening for prostate cancer: a guidance statement from the Clinical Guidelines Committee of the American College of Physicians. Ann Intern Med..

[16] Tong Y, Ru B, Zhang J (2018). miRNACancerMAP: an integrative web server inferring miRNA regulation network for cancer. Bioinformatics.

[17] Cheng G, Song Z, Liu Y, Xiao H, Ruan H, Cao Q, Wang K, Xiao W, Xiong Z, Liu D (2020). Long noncoding RNA SNHG12 indicates the prognosis of prostate cancer and accelerates tumorigenesis via sponging miR-133b. J Cell Physiol.

[18] Chen Z, Zhen M, Zhou J (2019). LncRNA BRE-AS1 interacts with miR-145-5p to regulate cancer cell proliferation and apoptosis in prostate carcinoma and has early diagnostic values. Biosci Rep..

[19] Schmitt AM, Chang HY (2016). Long noncoding RNAs in cancer pathways. Cancer Cell.

[20] Jahangirimoez M, Medlej A, Tavallaie M, Mohammad SB (2020). Hsa-miR-587 regulates TGFbeta/SMAD signaling and promotes cell cycle progression. Cell J.

[21] Ren Y, Dong J, He P, Liang Y, Wu L, Wang J, Chu B (2020). miR-587 promotes cervical cancer by repressing interferon regulatory factor 6. J Gene Med..

[22] Guo J, Lin Y, Wei J, Zhang X, Yang C, Sun J, Zhang Y, Hu G, Li J (2019). Diagnostic value of serum MiR-587 in patients with metabolic syndrome. Clin Lab..

[23] Dai Z, Popkie AP, Zhu WG, Timmers CD, Raval A, Tannehill-Gregg S, Morrison CD, Auer H, Kratzke RA, Niehans G (2004). Bone morphogenetic protein 3B silencing in non-small-cell lung cancer. Oncogene.

[24] Zhou T, Yu L, Huang J, Zhao X, Li Y, Hu Y, Lei Y (2019). GDF10 inhibits proliferation and epithelial-mesenchymal transition in triple-negative breast cancer via upregulation of Smad7. Aging (Albany NY).

[25] Cheng CW, Hsiao JR, Fan CC, Lo YK, Tzen CY, Wu LW, Fang WY, Cheng AJ, Chen CH, Chang IS (2016). Loss of GDF10/BMP3b as a prognostic marker collaborates with TGFBR3 to enhance chemotherapy resistance and epithelial-mesenchymal transition in oral squamous cell carcinoma. Mol Carcinog.

[26] Zhou B, Yi F, Chen Y, Li CH, Cheng YS, Yang K (2020). Reduced long noncoding RNA PGM5-AS1 facilitated proliferation and invasion of colorectal cancer through sponging miR-100-5p. Eur Rev Med Pharmacol Sci.

